# The Mediating Role of Forgiveness and Self-Efficacy in the Relationship Between Childhood Maltreatment and Treatment Motivation Among Malaysian Male Drug Addicts

**DOI:** 10.3389/fpsyg.2022.816373

**Published:** 2022-03-11

**Authors:** Loy See Mey, Rozainee Khairudin, Tengku Elmi Azlina Tengku Muda, Hilwa Abdullah @ Mohd Nor, Mohammad Rahim Kamaluddin

**Affiliations:** ^1^Department of Psychology, Jeffrey Cheah School of Medicine and Health Sciences, Monash University Malaysia, Subang Jaya, Malaysia; ^2^Faculty of Social Sciences and Humanities, Centre for Research in Psychology and Human Well-Being, Universiti Kebangsaan Malaysia, Bangi, Malaysia; ^3^PERMATApintar National Center, Universiti Kebangsaan Malaysia, Bangi, Malaysia

**Keywords:** forgiveness, self-efficacy, childhood maltreatment, treatment motivation, drug addicts

## Abstract

Studies have reported high rates of childhood maltreatment among individuals with drug addiction problems; however, investigation about the potentially protective factors to mitigate the effects of maltreatment experiences on motivation to engage in addiction treatment has received less attention. This study aims at exploring the mediating effects of forgiveness and self-efficacy on the association between childhood maltreatment and treatment motivation among drug addicts. A total of 360 male drug addicts (mean age = 33.34, *SD* = 7.25) were recruited from three mandatory inpatient rehabilitation centers in Malaysia. Participants completed a package of self-report questionnaires including measures of childhood maltreatment experiences, forgiveness, self-efficacy, and motivation for treatment. The analysis conducted using the structural equation model (SEM) revealed that childhood maltreatment significantly predicted lower treatment motivation, while forgiveness and self-efficacy played a fully mediating role regarding the effect of childhood maltreatment on treatment motivation. In conclusion, these findings suggest that combining the element of forgiveness and self-efficacy in treatment programs appears to benefit the drug addicts with childhood maltreatment history.

## Introduction

Exposure to maltreatment during childhood has long been associated with an increased risk of subsequent psychological and behavioral problems across the human lifespan including mood disorders (Norman et al., 2012; Lippard and Nemeroff, 2020), depression (Dhamayanti et al., 2020), post-traumatic stress disorder (McLaughlin et al., 2017; Schuck and Widom, 2019), borderline personality disorder (Mainali et al., 2020), adult criminality (Minh et al., 2013; Kim et al., 2016), and drug and substance abuse (Elwyn and Smith, 2013; Mandavia et al., 2016). Studies have demonstrated that maltreatment histories are relatively common among individuals receiving treatment for substance and drug abuse (Funk et al., 2003; Banducci et al., 2014; Loy et al., 2020a). However, most studies on the effects of childhood maltreatment among drug addicts are often focused on behavioral consequences and psychological risk factors associated with maltreatment, yet less is known about how the experience of maltreatment in the early stage impacts the motivation among drug addicts entering substance abuse treatment, which can be considered as a significant factor to influence the treatment outcomes and service provision.

Furthermore, studies indicated that not all children with maltreatment experience grow up to abuse drugs or substances (Simpson and Miller, 2002). Therefore, the mechanism regarding the pathways between childhood maltreatment and subsequent addiction, which might influence the later treatment engagement to abstain from drug use, remains unclear. Moreover, in Malaysia, various efforts have been made by the government to combat illicit drugs over the past decades; however, the continued failure of the war on drugs with increasing number of drug addicts being reported each year and high relapse rate following treatment has aroused controversies surrounding the effectiveness of government agencies and local leaders in addressing the country’s drug addiction problem. Therefore, examining the etiologies, such as early maltreatment history that might explain the onset of drug use, would be essential for refining the existing treatment program that lacks tackling the issue of adverse experience, particularly for drug addicts who have experienced adversity in early life. In short, interventions that can provide support to drug addicts’ unresolved childhood issues might help to increase their motivation to remain in treatment and further reduce their risk of relapse and improve their adaptive functioning after discharge from the rehabilitation center.

Rehabilitation programs are essential for recovery among drug addicts. However, research indicated that a high number of drug addicts receiving treatment failed to stay throughout the rehabilitation programs or experienced relapse due to adverse effects such as anxiety, anger, shame, and guilt (McKay, 2011; McGaffin et al., 2013; Serafini et al., 2016). Moreover, several studies have pointed out the use of the drug as a coping mechanism to eliminate the overwhelming negative emotions in drug addicts (Baker et al., 2004; Webb et al., 2006; Blevins et al., 2014). In view of the association between drug abuse, negative emotions, and treatment failure; forgiveness, which was well supported by literature to demonstrate a significant relationship with mental wellbeing and positive emotional states (Ricciardi et al., 2013; Raj et al., 2016; Toussaint et al., 2016; Long et al., 2020), has been hypothesized and proven to play a role in addiction and recovery (Worthington et al., 2006; Scherer et al., 2011).

Although the definition of forgiveness varied by the scholars who defined it, however, most definitions agreed that forgiveness refers to a decrease in negative feelings, thoughts, and behaviors while being able to cultivate positive or neutral emotions toward the wrongdoer (Enright, 1996; Maynard et al., 2016; Worthington et al., 2016). In relation to the context of trauma, a higher level of childhood maltreatment was often associated with a lower level of forgiveness (Guloglu et al., 2016; Arslan, 2017). Moreover, studies indicated that individuals who have experienced negative life events at an early age are predisposed to develop more maladaptive thoughts and behavioral dysfunction, which might impede their coping skills and strategies later in life (Pears and Fisher, 2005; Kim and Cicchetti, 2010; Milojevich et al., 2018). Furthermore, it seems reasonable to expect that drug use would be the most convenient option for someone with poor coping skills to deal with negative emotions. Therefore, in view of the positive effects shown by forgiveness, this element can be considered as an effective coping strategy to buffer against the negative impacts of traumatic experience, to improve the mental wellbeing (e.g., anger, guilt, and depression), and, finally, to improve the drug treatment outcomes. In other words, forgiveness could be a positive coping mechanism in lieu of drugs.

The concept of self-efficacy, which originated from Bandura’s Social Learning Theory, referred to a person’s beliefs in their capacity to use resources and skills to accomplish certain tasks (Bandura, 1993). In the context of drug abuse, abstinence self-efficacy, which is considered as a specific form of self-efficacy to examine the ability among drug addicts to abstain from drug use, has been identified as a significant intrapersonal resource to predict substance use (McKay et al., 2004; Majer et al., 2016) and also as a crucial component to improve drug treatment outcomes (Litt et al., 2008; Kadden and Litt, 2011) and future abstinence (Chavarria et al., 2012). However, in drug treatment research, an area in which knowledge is currently lacking is the association between drug addicts’ motivation when entering drug treatment in relation to their level of self-efficacy. Moreover, existing research in this area has tended to focus on abstinence self-efficacy (Kelly and Greene, 2014; Majer et al., 2015) rather than to explore the role of general self-efficacy which covers the overall beliefs in drug addict’s ability to succeed in treatment.

Studies have indicated that self-efficacy played a role as an effective predictor of children’s subsequent development (Bandura, 1993; Tsang et al., 2012). Moreover, in the context of child abuse, childhood maltreatment is associated with lower self-efficacy and various health problems in adulthood (Sachs-Ericsson et al., 2011; Taylor et al., 2016; Basu et al., 2017). According to the attachment theory, childhood maltreatment negatively affects the formation of secure attachments among children which later poses a challenge to the development of self-efficacy beliefs throughout their lives (Riggs, 2010) which, in turn, can affect one’s self-regulation of motivation to accomplish something later in life (Tsang et al., 2012). In short, the adverse experience in the early years can negatively impact the cognitions about the self and the control one can exert over his or her own functioning.

Significantly, the evidence of individuals’ differences in negative outcomes, such as the development of drug use problems in response to childhood maltreatment, implies an indirect pathway between the relationship of maltreatment experience and drug addiction (Simpson and Miller, 2002; Rutter, 2007; Wahab et al., 2021). In other words, other factors may play a role in explaining the mechanism underlying this relationship. In addition, in view of the inconsistent findings reported by previous studies between the association of maltreatment experience and drug treatment motivation (Battjes et al., 2003; Rosenkranz et al., 2012; Lu et al., 2017), the purpose of this study was to examine the relationship between childhood maltreatment and motivation among drug addicts entering treatment and the factors that have the potential to explain the relationship between the two variables.

In view of the impacts of childhood maltreatment on the formation of forgiveness and self-efficacy and the significant role as coping strategy played by both factors (Witvliet and McCullough, 2007; Cieslak et al., 2008), this study hypothesized that the association between childhood maltreatment and treatment motivation among drug addicts would be mediated by individual differences in forgiveness and self-efficacy, with higher levels of forgiveness and self-efficacy predicting higher levels of motivation in treatment. In addition, there is evidence that different types of childhood maltreatment experiences may yield specific pathways to the development of negative outcomes (Infurna et al., 2016). Thus, this study further examined the predictive value of different forms of childhood maltreatment, which included emotional, physical, sexual abuses, and emotional and physical neglect on motivation as well as its association with forgiveness and self-efficacy.

## Materials and Methods

### Participants and Procedure

The study protocol was approved by the National Anti-Drugs Agency (AADK) of Malaysia. All participants were recruited from three mandatory drug rehabilitation centers managed by AADK that comes under the supervision of the Malaysia Home Ministry that provides free treatment and rehabilitation programs to the individual who has been confirmed as a drug addict. First, a pilot study was conducted by recruiting 160 inpatient drug addicts from a drug rehabilitation center, where 144 questionnaires were completed and used for the analysis. Next, in view of the gender ratio of drug addicts which was close to 40 males per 1 female in Malaysia according to the statistics provided by AADK, a total of 380 male inpatient drug addicts entering substance abuse treatment were selected through a simple random sampling. However, after excluding the poorly completed questionnaires and missing data, 360 samples remained to use for the analysis. A briefing session regarding the ethical measures and research objectives was conducted at the beginning, and informed consent was requested from each participant to use the information for research purposes. The average age of the sample was 33.34 years (*SD* = 7.25), and participants were predominately Malay (96.4%) and single (53.1%). All participants have gone through evaluations and assessments and were diagnosed with substance use disorder (SUD) by psychiatrists and narcotic officers under AADK before they were admitted to the rehabilitation centers to receive treatment. In addition, several different drugs were reported by the participants throughout their drug use histories which encompassed opioids, ecstasy, amphetamines, cocaine, marijuana, and other types of psychotropic drugs. All recruited participants were with reading and comprehension abilities to take part in the research.

### Measures

#### Childhood Maltreatment

Childhood maltreatment was examined using the 28 items of the Childhood Trauma Questionnaire Short Form (CTQ-SF). CTQ-SF is a retrospective self-administered screening device used to detect histories of childhood abuse and neglect. It is the most commonly used and valid assessment tool to enable the identification of five dimensions of childhood maltreatment experiences, namely, emotional abuse (EA), physical abuse (PA), sexual abuse (SA), emotional neglect (EN), and physical neglect (PN) (Bernstein et al., 2003). The original version of CTQ-SF has shown sufficient psychometric properties across different settings (Bernstein and Fink, 1998; Gerdner and Allgulander, 2009). Meanwhile, the Malay version of CTQ-SF (M-CTQ-SF), which exhibited good performance in reliability and validity, and cultural equivalence in the Malaysian population, was used in this study (Loy et al., 2020b). Participants were asked to report the frequency of their maltreatment experience during their first 16 years of life by using a 5-point Likert-type response, ranging from 1 = never to 5 = very often. Items are generally phrased in a non-evaluative manner, given as follows: “People in my family said hurtful or insulting things to me” for emotional abuse and “My family was a source of strength and support” for emotional neglect. Overall, the raw and total scores of CTQ-SF examine the separate and combined effects of multiple forms of childhood abuse and neglect experiences. The Cronbach’s alpha coefficient of the total M-CTQ-SF was 0.88, while the alpha coefficient for the five subscales ranged from 0.61 for sexual abuse to 0.81 for emotional abuse in the current sample.

#### Motivation in Treatment

The Circumstances, Motivation, and Readiness (CMR) Scales were used to examine the drug addicts’ motivation in treatment. The CMR is a self-report measure with 18 items scoring on a 5-point Likert-type response ranging from 1 = strongly disagree to 5 = strongly agree. The subscale of Circumstances refers to the external factors that influence an individual to seek treatment, the Motivation subscale refers to an individual’s inner factors for change, whereas the Readiness subscale assesses an individual’s perceived need for treatment in order to change (De Leon et al., 1994). Besides, potential total scores for CMR range from 0 to 90 with higher scores indicating higher motivation and readiness for treatment. Overall, CMR demonstrated adequate total score reliability with Cronbach’s alpha coefficient ranging from 0.70 to 0.80 across a wide variety of substance-using populations (De Leon et al., 2000; Melnick et al., 2014). Meanwhile, the Cronbach’s alpha of the total scores of CMR was 0.843 in this sample.

#### Forgiveness

The Heartland Forgiveness Scale (HFS) was used to measure an individual’s dispositional forgiveness of themselves, others, and situations. The HFS is an 18-item self-report measure scoring on a 7-point Likert-type response ranging from 1 = almost always false of me to 7 = almost always true of me. Participants were asked to report their typical responses to the negative events which occur due to their own actions (e.g., “I hold grudges against myself for negative things I’ve done”), the actions of others (e.g., “I continue to be hard on others who have hurt me”), or circumstances out of their control (e.g., “It’s really hard for me to accept negative situations that aren’t anybody’s fault”). Besides, total HFS scores range from 18 to 126 with higher scores indicating higher levels of forgiveness. Previous research reported adequate psychometric properties with Cronbach’s alpha coefficient ranging from 0.86 to 0.87 for the total HFS, while 0.72–0.82 for the subscales (Thompson et al., 2005). In this sample, the Cronbach’s alpha of HFS was 0.825.

#### Self-Efficacy

The General Self-Efficacy Scale (GSES) is a 10-item self-report measure that was used to assess an overall sense of self-belief in coping with various difficult demands in life (Schwarzer and Jerusalem, 1995). A sample item included: “It is easy for me to stick to my aims and accomplish my goals.” Participants were asked to rate their agreement on each item on a 4-point Likert-type response ranging from 1 = not at all true to 4 = always true. The GSES sum score ranges from 10 to 40 with higher scores indicating greater self-efficacy. Besides, this scale has been shown valid and reliable in numerous studies, with good internal consistency ranging from 0.75 to 0.94 (Schwarzer et al., 1997; Luszczynska et al., 2005); meanwhile, the Cronbach’s alpha coefficient of GSES in this sample was 0.908.

#### Statistical Analysis

Overall, several data analyses were conducted to examine the associations between childhood maltreatment, treatment motivation, forgiveness, and self-efficacy. First, the exploratory factor analysis (EFA) was carried out *via* IBM-SPSS 22 using the data from the pilot study to explore the usefulness of items that measure the respective constructs being studied. Certain items with poor factor loading were being removed based on the EFA results (McNeish, 2017). Thereafter, in order to investigate the validity of the model, 360 sets of field data (collected *via* the final version of questionnaires which is being constructed using the results of EFA) were used to conduct the confirmatory factor analysis (CFA) using IBM-SPSS AMOS 22. Following CFA, associations between the variables were examined with the scores of the variables being standardized into *z*-values. Furthermore, the structural equation modeling (SEM) was performed to examine the interrelationships among the constructs in this study. Finally, the mediation effects of forgiveness and self-efficacy were assessed using 5,000 bootstrapped samples with a 95% CI.

## Results

### Validity and Reliability

Prior to performing the SEM for hypothesis testing, CFA was conducted to examine the construct validity, convergent validity, and discriminant validity of all constructs that were being researched in this study (Hair et al., 2006). The three constructs, namely, childhood maltreatment, forgiveness, and treatment motivation, being studied were second-order constructs with complicated measurement model; therefore, the CFA for each measurement model was assessed separately, and all models were combined to perform a pooled-CFA after all constructs achieved the respective thresholds of validity and reliability in individual assessment (Awang et al., 2018). The results of individual CFA assessment for all constructs were presented in Supplementary Appendix.

**FIGURE 1:**
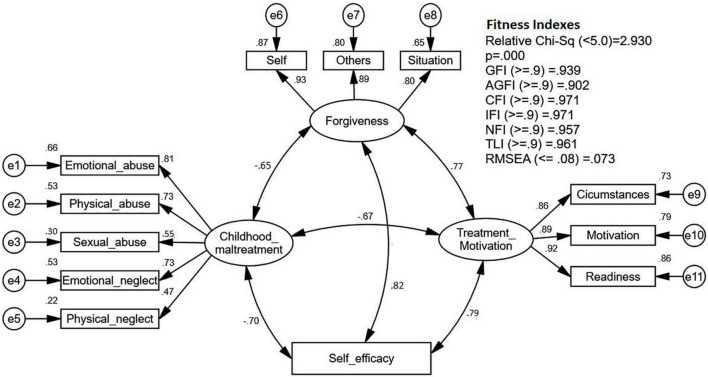
The Results of Confirmatory Factor Analysis (CFA).

Thereafter, in pooled-CFA assessment, all second-order constructs that have been validated were simplified into first-order constructs. First, the construct validity was assessed by looking at the fitness indexes of the model. According to the CFA outputs, as presented in Figure 1, the fitness indexes have achieved the requirement of construct validity with the absolute fit (RMSEA = 0.073) less than 0.08 and the parsimony fit index (chi^2^/df = 2.930) below 5, while all the other indices (e.g., GFI, CFI, IFI, NFI, TLI) were higher than 0.9 (Awang et al., 2018). Moreover, the factor loadings for all items were above 0.6, which indicated the unidimensionality of the model (Byrne, 2016) except for SA and PN with factor loadings below the cutoff point. However, the two items were retained due to the suggestion that factor loading above 0.4 in CTQ-SF was acceptable (Bernstein et al., 1994).

The average variance extracted (AVE), which determines the convergent validity, and CR, which determines the composite reliability for all latent constructs, are presented in Table 1. Because the values for both AVE and CR were computed using the factor loadings, the AVE for the construct of childhood maltreatment was unsatisfactory (0.45), while the AVE for other latent constructs was above 0.5, which indicated a high convergent validity (Byrne, 2016). However, since the composite reliability of childhood maltreatment was above 0.6, its convergent validity is still considered adequate (Fornell and Larcker, 1981). Besides, concerning the discriminant validity, AVE for each latent construct in the model was greater than its respective square root of correlation value with other constructs as presented in Table 1 (AVE > *r*^2^); thus, the discriminant validity of all latent constructs was accomplished (Byrne, 2010). Furthermore, the correlation value (*r*) between self-efficacy with its respective constructs was all below 0.85, which indicated that the model was free from the multicollinearity problem. Therefore, it can be concluded that the construct validity, convergent validity, and discriminant validity of all constructs in this study have been achieved.

**TABLE 1:** Discriminant validity index summary.

Construct	Composite reliability	Childhood maltreatment	Forgiveness	Treatment motivation
Childhood maltreatment	0.80	**0.45**		
Forgiveness	0.91	0.42	**0.77**	
Treatment motivation	0.91	0.44	0.59	**0.79**

*Bold values indicate average variance extracted (AVE).*

### Relationship Analysis

Findings from preliminary analysis demonstrated that all variables in this sample have adequate internal reliability, ranging from 0.80 to 0.89. Thereafter, Pearson correlation was conducted in exploring the relationship among childhood maltreatment, forgiveness, self-efficacy, and treatment motivation. Childhood maltreatment was negatively correlated with treatment motivation (*r* = 0.562, *p* < 0.01), forgiveness (*r* = –0.638, *p* < 0.01), and self-efficacy (*r* = –0.612, *p* < 0.01). In addition, treatment motivation indicated a positive relationship with forgiveness (*r* = 0.810, *p* < 0.01) and self-efficacy (*r* = 0.812, *p* < 0.01). Furthermore, the inter-correlations between the total score of CTQ-SF with the five maltreatment subscales were found to be significant. Among the five subscales, EN and SA indicated the highest and lowest correlations with the total CTQ-SF, respectively (Table 2).

**TABLE 2:** Descriptive statistics and correlation results of variables.

Construct	Descriptive statistics									
	Mean	*SD*	CTQ-SF	PA	EN	EA	SA	PN	HFS	GSE
CTQ-SF	40.81	12.11	1							
PA	8.53	3.22	0.801	1						
EN	11.21	4.23	0.823	0.532	1					
EA	8.50	3.54	0.833	0.652	0.523	1				
SA	5.29	2.23	0.482	0.329	0.147	0.298	1			
PN	6.21	2.81	0.611	0.238	0.495	0.324	0.162	1		
HFS	64.22	28.3	–0.638	–0.479	–0.572	–0.557	–0.167	–0.269	1	
GSE	29.73	6.73	–0.612	–0.501	–0.544	–0.523	–0.155	–0.283	0.862	1
CMR	41.44	13.9	–0.562	–0.424	–0.521	–0.447	–0.222	–0.256	0.810	0.812

*All correlation values are significant at the 0.01 level (2-tailed).*

*CTQ-SF, total score of childhood maltreatment; PA, physical abuse; EN, emotional neglect; EA, emotional abuse; SA, sexual abuse; PN, physical neglect; HFS, forgiveness; GSE, self-efficacy; CMR, treatment motivation.*

### Model Analysis

**FIGURE 2:**
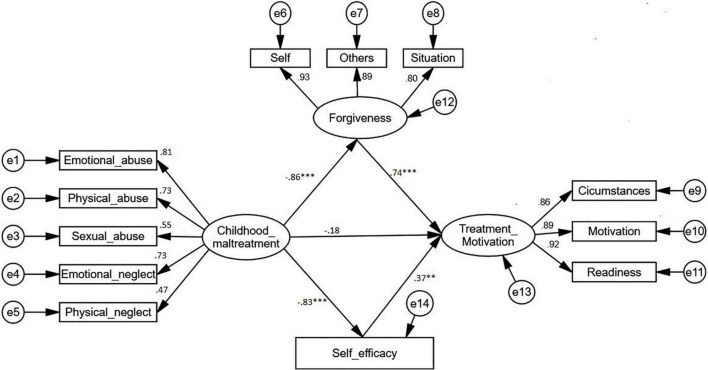
The standardized regression path coefficient between constructs. ^***^p ≤ 0.001, ^**^p ≤ 0.05.

After all the constructs have been validated, SEM was followed to test the interrelationships among the constructs in the model. Overall, the results of the regression path coefficient (β) which reflected the predictive effects of exogenous constructs on the endogenous constructs were presented in Figure 2 with the interpretation in Table 3. Childhood maltreatment was a negative predictor of forgiveness (β = –0.861, *p* < 0.001) and self-efficacy (β = –0.831, *p* < 0.001), yet it did not significantly predict treatment motivation (β = –0.178, *p* = 0.678) in the mediation model. Therefore, the association between childhood maltreatment, forgiveness, and treatment motivation was continued to assess in the direct model. Moreover, treatment motivation was significantly predicted by forgiveness (β = 0.740, *p* < 0.001) and self-efficacy (β = 0.373, *p* < 0.032).

**TABLE 3:** The regression path coefficient.

Path	β	p	95% bootstrap BC CI	
			LB	UB
Childhood maltreatment → Forgiveness	–0.861	0.001	–2.32	–1.62
Childhood maltreatment → Self-efficacy	–0.831	0.001	–0.243	–1.32
Childhood maltreatment → Treatment motivation	–0.178	0.678	–0.186	0.133
Forgiveness → Treatment motivation	0.740	0.001	0.261	0.587
Self-efficacy → Treatment motivation	0.373	0.032	0.037	0.251

### Mediating Analysis

**FIGURE 3:**
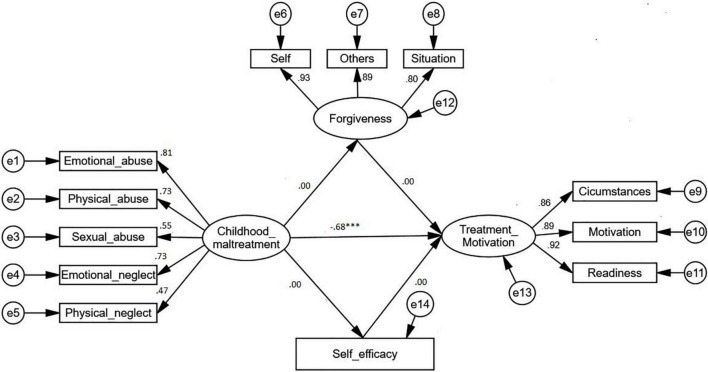
The direct model. ^***^p ≤ 0.001.

In order to examine whether forgiveness plays a mediator role between childhood maltreatment and treatment motivation, the bootstrap procedure involving both the mediation model and direct model was utilized. Results from these two models were used to assess for the mediation effect of forgiveness (Table 4). In the direct model, all the indirect paths were constrained to zero in order to eliminate the mediator effect in the model (Figure 3). As a result, without the interference of the mediator, treatment motivation was significantly predicted by childhood maltreatment (β = –0.678 *p* < 0.001). Taken together, these results supported the predictive role of childhood maltreatment in treatment motivation *via* forgiveness and self-efficacy, with forgiveness and self-efficacy fully mediating this association.

**TABLE 4:** Results of the direct model.

Path	β	*p*	95% bootstrap BC CI	
			LB	UB
**Direct model**				
Childhood maltreatment → Treatment motivation	–0.68	0.001	–1.341	0.928

### The Effects of Different Forms of Childhood Maltreatment

**FIGURE 4:**
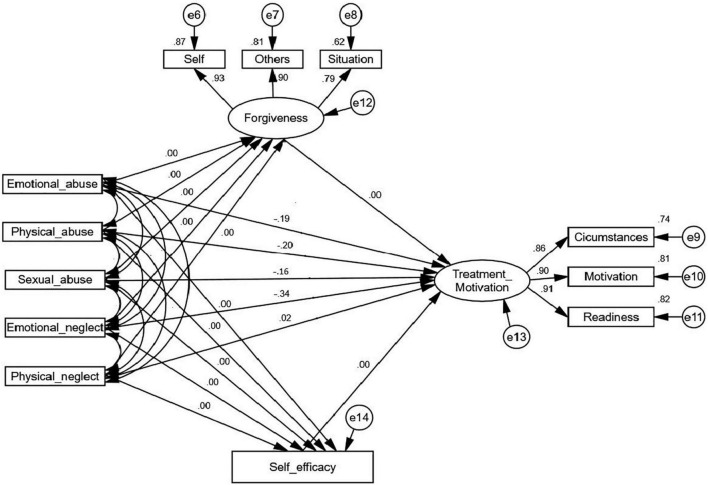
The direct model of various forms of maltreatment. All indirect paths constrained to 0.

Overall, this study aimed to examine the association between childhood maltreatment and treatment motivation among drug addicts; thus, besides investigating the overall effects of childhood maltreatment as an individual variable on its endogenous constructs, the predictive value of various forms of maltreatment from the overall maltreatment in the model was further assessed to attain a more holistic data. As presented in Figure 4, the outcomes produced by SEM were consistent with the previous results when replacing the total scores of maltreatment with different forms of maltreatment. In other words, four forms of maltreatment, namely, EA, PA, SA, and EN, were significantly related to forgiveness and self-efficacy, and they played a predictive role in treatment motivation in the direct model (Table 5). These results further suggested that a strong association exists between the various forms of maltreatment with forgiveness and treatment motivation among drug addicts. Furthermore, it also supported the full mediating role of forgiveness in the relationship between various forms of maltreatment and treatment motivation.

**TABLE 5:** Results of mediation effect of forgiveness and self-efficacy on various forms of maltreatment and treatment motivation.

Path	β	*p*
**Direct model**		
Emotional abuse → Treatment motivation	–0.19	0.003
Physical abuse → Treatment motivation	–0.20	0.001
Sexual abuse → Treatment motivation	–0.16	0.046
Emotional neglect → Treatment motivation	–0.34	0.001
Physical neglect → Treatment motivation	–0.02	0.546
**Mediation model (Forgiveness)**		
Emotional abuse →Forgiveness	–0.289	0.001
Physical abuse →Forgiveness	–0.184	0.001
Sexual abuse →Forgiveness	–0.171	0.042
Emotional neglect →Forgiveness	–0.365	0.001
Physical neglect →Forgiveness	0.049	0.685
Forgiveness → Treatment motivation	0.670	0.001
**Mediation model**		
Emotional abuse →Self-efficacy	–0.280	0.001
Physical abuse → Self-efficacy	–0.167	0.002
Sexual abuse → Self-efficacy	0.026	0.126
Emotional neglect → Self-efficacy	–0.336	0.001
Physical neglect → Self-efficacy	0.025	0.224
Self-efficacy → Treatment motivation	0.814	0.001

## Discussion

Child maltreatment that included various forms of abuse and neglect is a significant social problem that affects all races, ethnicities, and socioeconomic groups with severe lifelong consequences. Research has highlighted the link between childhood maltreatment and later engagement in high-risk behaviors, such as substance and drug abuse (Mandavia et al., 2016; Yen et al., 2021; Lim et al., 2021), followed by different kinds of mental difficulties that pose threat to the motivation of abstaining from addictive behaviors (Grella and Joshi, 2003; Rosenkranz et al., 2012). Therefore, this study aimed to alleviate the negative impacts of childhood maltreatment on motivation to engage in treatment programs among drug addicts. To this end, this study examined the potential variables that were deemed effective in reducing the negative emotions resulting from past adverse experiences in a healthy way. Furthermore, both forgiveness and self-efficacy, which were considered as positive elements with a strong association with resilience (Srivastava, 2011; Kelly, 2018; Bikar et al., 2021), have been investigated for their mediating effects on the association between childhood maltreatment and treatment motivation in drug addicts.

Overall, the results of this study were consistent with previous research that demonstrated a significant association between childhood maltreatment and treatment motivation among drug addicts undergoing inpatient treatment programs. The negative association between both overall childhood maltreatment and different forms of maltreatment with treatment motivation supported and extended the previous research that the childhood adverse personal histories could undermine the treatment engagement and recovery process (Sacks et al., 2008; Lu et al., 2017). Moreover, the present results implied the role of a drug as an unhealthy alternative for the addicts with adverse histories to escape from pain and emotional distress. Previous research has indicated the myriad challenges encountered by addicts with histories of abuse in drug treatment including negative psychological functioning (Sacks et al., 2008) and greater severity of anxiety that co-occurred with strong feelings of shame, anger, and self-blame (Bennett et al., 2005; Rosenkranz et al., 2012), which could pose formidable threats to their willingness to abstain from drug use. Furthermore, the findings of Grella and Joshi (2003) indicated that a more intensive level of care in treatment required by drug addicts with histories of abuse also implied the negative impacts of childhood maltreatment on treatment motivation.

Moreover, findings from this study indicated that childhood maltreatment is a significant predictor of forgiveness, consistent with previous studies that reported a negative association between maltreatment histories and forgiveness (Snyder and Heinze, 2005; Arslan, 2017). Various negative outcomes on physical and mental health are well documented for those who experience childhood maltreatment (Currie and Widom, 2010; Norman et al., 2012; Tharshini et al., 2021), thereby potentially affecting their social functioning, such as lack of engagement and support from families in the growing up process (Lamis et al., 2014). Moreover, Müller et al. (2019) demonstrated the negative impacts of poor social functioning as a result of childhood maltreatment, which includes difficulties making connecting relationships and insensitivity toward others, which may thereby affect their development of forgiveness (Müller et al., 2019). Furthermore, considering the nature of abusive families, maltreated children were prone to believe that they cannot rely on others for care and support, which may later develop an insecure attachment style (Ainsworth, 1989). Research indicated that individuals with insecure attachment have more difficulty repairing the relationship after an offense occurs (McCullough et al., 1998) and as well demonstrated a lower level of forgiveness (Burnette et al., 2009). To sum up the literature, an individual’s forgiveness may indirectly be affected by childhood maltreatment experiences, and it seems logical to expect that individuals with maltreatment histories might face various challenges in the development of forgiveness, supported by findings from this study.

First, the indirect effect analysis demonstrated the full mediation effect of forgiveness in the relationship between childhood maltreatment and treatment motivation. In other words, the results supported the role of forgiveness as a mitigating factor in alleviating the effect of childhood maltreatment on treatment motivation among drug addicts. Thus, forgiveness might be an effective element in helping drug addicts in the face of childhood adverse experiences. Research has indicated that childhood maltreatment was the predictor of shame proneness, and drug use may work as the mechanism to cope with negative emotions (O’Connor et al., 1994; Rahim and Patton, 2015). Besides, previous studies have demonstrated higher levels of anger among drug addicts (Lin et al., 2004), which may function as a defense mechanism against the feeling of shame and guilt associated with their addiction behavior (Worthington et al., 2006). Thus, it seems reasonable to expect a cyclical relationship between negative emotions and drug addiction. Therefore, an introduction of a positive element, such as forgiveness, may help in alleviating negative feelings, thereby reducing the probability of drug use as a coping mechanism, and may further help in increasing their motivation to engage in treatment. Moreover, in addition to the positive effects of forgiveness on psychological and physical wellbeing as having been supported by previous research (Toussaint et al., 2016; Long et al., 2020), forgiveness is as well considered an effective element to cope with maltreatment experiences in the context of trauma (Freedman and Enright, 2017). Taken together, the literature supported the positive effect of forgiveness on one’s wellbeing, which is consistent with the present findings indicating its full mediating role in the association between childhood maltreatment and treatment motivation among drug addicts.

On the contrary, in view of earlier research that has identified deficits in self-efficacy as a potentially negative outcome of early maltreatment experiences (Sachs-Ericsson et al., 2011; Singer et al., 2016), this study sought to support a relationship between childhood maltreatment and self-efficacy. As a result, the negative association between the two constructs was found in the SEM analysis. Meanwhile, the significant association between self-efficacy and treatment motivation demonstrated that the efforts in this study to explore the mediating effect of self-efficacy between childhood maltreatment and treatment motivation were deemed successful. In other words, this finding indicated that negative experiences in childhood lowered the level of treatment motivation *via* a negative self-efficacy. Thus, consistent with previous studies, the results of this study suggested that childhood maltreatment experiences predict a low level of self-efficacy after decades.

Nevertheless, the indirect effect analysis showed that the association between self-efficacy and treatment motivation was statistically significant but not at the 0.001 level. Research has indicated that drug addicts with maltreatment histories reported a significantly higher number of lifetime addiction and treatment admission (Westermeyer et al., 2001; Khoury et al., 2010), which implies a high relapse rate after they were successfully recovered from addiction and were discharged from rehabilitation centers. Consequently, the high failure records to abstain from drug use might either negatively affect their sense of self-efficacy or strengthen their beliefs in their capacity to complete the treatment program and recover from addiction once again. Therefore, it seems reasonable to expect that some drug addicts involved in this sample have higher levels of self-efficacy. Furthermore, this sample reported high levels of self-efficacy on average (mean = 29.6) (Table 1), with the scale score ranging from a minimum of 10 to a maximum of 40, thereby causing a weaker effect for self-efficacy in this equation. Overall, the mediating results of this study suggested the critical role of forgiveness in drug treatment, while improving self-efficacy may be beneficial for drug addicts to abstain from drug use.

## Limitations and Future Research

There were several methodological limitations to be considered when interpreting the results of this study. First, this sample was not ethnic and gender diverse. Previous research has indicated the gender, ethnic, and cultural differences in child maltreatment and its consequences (Lee et al., 2012; Asscher et al., 2015; Lansford et al., 2015). Moreover, several studies have pointed out the gender and cultural diversity in forgiveness (Toussaint and Webb, 2005; Paz et al., 2008) and self-efficacy (Klassen, 2004; Wang et al., 2019). However, the majority of respondents in this study were Malay males (96.4%), which may limit the findings to be generalized to a more diverse sample. Therefore, there is a need for future research to identify male and female samples of drug addicts from different ethnic groups that may be directly compared on the variables of various forms of childhood maltreatment and its effects on forgiveness, self-efficacy, and treatment motivation, which may thereby provide useful information regarding unique and common elements of treatment programs for drug-addicted male and female subjects.

Moreover, the study was conducted using a cross-sectional approach in which the data of childhood maltreatment, forgiveness, self-efficacy, and treatment motivation were collected at one point in time. However, several investigated variables are likely to fluctuate over time. For example, Gecas (1989) pointed out the dynamic nature of self-efficacy which may change over the life course depending on the immediate social context (Gecas, 1989). Moreover, previous research has demonstrated the relationship between active participation in addiction treatment programs with greater self-efficacy (Ilgen et al., 2005; McKellar et al., 2008). Thus, this might explain the average high levels of reported self-efficacy in this study, which might probably decrease after the completion of treatment. In addition, McCullough et al. (2003) also underlined the fluctuation of forgiveness over time (McCullough et al., 2003). Consequently, these instability factors limited the generalizability of the results. Thus, future research may focus on capturing fluctuations in these dynamic variables that might be related to treatment motivation and dropout in drug addicts by conducting a longitudinal study.

In addition, the respondents were asked to recall their maltreatment experiences that occurred before the age of 16 years (Bernstein et al., 2003). Their reports regarding the adverse experience that happened to them in childhood may have been influenced by their current health conditions and many years of illicit drug use after a long period of time, which thereby may complicate their memories regarding the effects of childhood maltreatment on their intention to use drug and motivation to abstain from drug use. Moreover, research has demonstrated that individuals who reported poor health conditions may have a bias to recall their childhood maltreatment experiences (Edwards et al., 2001; Sheikh, 2018). Therefore, future research may pay more attention to the factors that might aggravate the recall bias such as the length and duration of the questionnaire or bias explanations regarding the study’s objective in order to obtain a more precise estimate of the prevalence of childhood maltreatment histories.

Finally, lower socioeconomic status has consistently been shown to be associated with a higher risk of child maltreatment (Drake and Pandey, 1996; Lefebvre et al., 2017) mainly because of the stress and conflict that arise between children and parents due to economic pressures. Therefore, children from low-income families are more likely to be exposed to child abuse and neglect; meanwhile, poverty, which is usually followed by unemployment and insufficient level of education attainment, might increase stress and the likelihood of abuse drugs in adulthood. In short, all these confounding variables may influence the respondents’ motivation to abstain from drug use which might pose a threat to the research reliability and validity. Thus, future research should attempt to validate the findings of this study with comparative data from different socioeconomic groups of drug addicts.

## Conclusion

Findings from this study highlighted the critical role of negative personal experiences developed early in life that appeared to carry over into adulthood and possibly incline the victims toward drug use to escape from painful memories or an attempt to reduce negative emotions associated with trauma, thereby negatively affecting their willingness to abstain from drug use or compromise their motivation in addiction treatment. Furthermore, the results demonstrated that forgiveness and self-efficacy played the role as protective factors to reduce the effects of childhood maltreatment experiences on treatment motivation. Moreover, the findings indicated that total scores of childhood maltreatment yielded similar results with different forms of maltreatment in relation to other investigated variables in this study, and supported and confirmed the co-occurrence across multiple types of maltreatment in an abusive family as has been found in earlier studies (Descartes et al., 2020). In addition, the mediating role of self-efficacy demonstrated that high self-efficacy may play a role in buffering the negative effects of childhood maltreatment on treatment motivation. Finally, forgiveness, by effectively reducing the negative emotions among drug addicts, may promote acceptance of self and further increase their motivation to engage in addiction treatment, thereby potentially preventing relapse. However, although forgiveness can be recommended as a mitigating element to assist individuals with a history of maltreatment, counselors or other professionals need to be careful when offering it. Recovery involving forgiveness is a difficult and long process, so avoid rushing to the outcomes and beware not to instill any kind of moral responsibility in victims to forgive their parents who have had hurt them.

## Data Availability Statement

The raw data supporting the conclusions of this article will be made available by the authors, without undue reservation.

## Author Contributions

LS: manuscript writing, data collection, and data analysis. MK: manuscript writing. RK, TT, and HA: data collection. All authors contributed to the article and approved the submitted version.

## Conflict of Interest

The authors declare that the research was conducted in the absence of any commercial or financial relationships that could be construed as a potential conflict of interest.

## Publisher’s Note

All claims expressed in this article are solely those of the authors and do not necessarily represent those of their affiliated organizations, or those of the publisher, the editors and the reviewers. Any product that may be evaluated in this article, or claim that may be made by its manufacturer, is not guaranteed or endorsed by the publisher.
