# Supplementary material for: The Mediating Role of Forgiveness and Self-Efficacy in the Relationship Between Childhood Maltreatment and Treatment Motivation Among Malaysian Male Drug Addicts
Source: Front Psychol. 2022 Mar 11;13:816373. doi: 10.3389/fpsyg.2022.816373 (PMC8963337; doi:10.3389/fpsyg.2022.816373)
Supplement: Supplementary file 1 [file Table_1.DOCX]

**Appendix A**

**Table A1. Results of CFA assessment for childhood maltreatment**

| **Construts** | **Item** | **Factor Loading** | **AVE** | **CR** |
| --- | --- | --- | --- | --- |
| Child maltreatment | EA | .79 | .429 | .786 |
|  | PA | .70 |  |  |
|  | SA | .55 |  |  |
|  | EN | .72 |  |  |
|  | PN | .46 |  |  |
| EA | CTQ3 | .60 | .417 | .739 |
|  | CTQ14 | .74 |  |  |
|  | CTQ18 | .57 |  |  |
|  | CTQ25 | .66 |  |  |
| PA | CTQ11 | .73 | .414 | .736 |
|  | CTQ12 | .64 |  |  |
|  | CTQ15 | .64 |  |  |
|  | CTQ17 | .55 |  |  |
| SA | CTQ20 | .68 | .434 | .693 |
|  | CTQ24 | .54 |  |  |
|  | CTQ27 | .74 |  |  |
| EN | CTQ5 | .62 | .487 | .825 |
|  | CTQ7 | .76 |  |  |
|  | CTQ13 | .70 |  |  |
|  | CTQ19 | .72 |  |  |
|  | CTQ28 | .68 |  |  |
| PN | CTQ1 | .67 | .463 | .721 |
|  | CTQ2 | .68 |  |  |
|  | CTQ6 | .69 |  |  |

Note. AVE = Average Variance Extracted, CR = Composite Reliability, EA = emotional abuse, PA = physical abuse, SA = sexual abuse, EN = emotional neglect, PN = physical neglect

**Table A2. Results of CFA assessment for forgiveness**

| **Construts** | **Item** | **Factor Loading** | **CR** | **AVE** |
| --- | --- | --- | --- | --- |
| Forgiveness | Forgiveness of self | .95 | .939 | .836 |
|  | Forgiveness of others | .94 |  |  |
|  | Forgivenss of situaion | .85 |  |  |
| Forgiveness of self | HFS1 | .96 | .968 | .859 |
|  | HFS3 | .97 |  |  |
|  | HFS5 | .95 |  |  |
|  | HFS2 | .89 |  |  |
|  | HFS6 | .86 |  |  |
| Forgiveness of others | HFS10 | .92 | .898 | .688 |
|  | HFS12 | .81 |  |  |
|  | HFS9 | .80 |  |  |
|  | HFS11 | .78 |  |  |
| Forgivenss of situaion | HFS14 | .92 | .915 | .730 |
|  | HFS16 | .95 |  |  |
|  | HFS18 | .77 |  |  |
|  | HFS13 | .76 |  |  |

Note. AVE = Average Variance Extracted, CR = Composite Reliability.

**Table A3. Results of CFA assessment for self-efficacy**

| **Construt** | **Item** | **Factor Loading** | **CR** | **AVE** |
| --- | --- | --- | --- | --- |
| Self-efficacy | GSE1 | .90 | .946 | .689 |
|  | GSE3 | .81 |  |  |
|  | GSE4 | .86 |  |  |
|  | GSE5 | .84 |  |  |
|  | GSE6 | .83 |  |  |
|  | GSE8 | .68 |  |  |
|  | GSE9 | .81 |  |  |
|  | GSE10 | .89 |  |  |

Note. AVE = Average Variance Extracted, CR = Composite Reliability

**Table A4. Results of CFA assessment for treatment motivation**

| **Konstruk** | **Item** | **Factor Loading** | **CR** | **AVE** |
| --- | --- | --- | --- | --- |
| Treatment motivation | Circumstances | .99 | .982 | .948 |
|  | Motivation | .95 |  |  |
|  | Readiness | .98 |  |  |
| **Subkonstruk** |  |  |  |  |
| Circumstances | CMR1 | .80 | .727 | .572 |
|  | CMR2 | .71 |  |  |
| Motivation | CMR7 | .80 | .895 | .632 |
|  | CMR8 | .69 |  |  |
|  | CMR9 | .82 |  |  |
|  | CMR10 | .89 |  |  |
|  | CMR11 | .76 |  |  |
| Readiness | CMR13 | .76 | .888 | .666 |
|  | CMR14 | .86 |  |  |
|  | CMR17 | .84 |  |  |
|  | CMR18 | .80 |  |  |

Note. AVE = Average Variance Extracted, CR = Composite Reliability
